# Electrical Resistivity and Joule Heating Characteristics of Cementitious Composites Incorporating Multi-Walled Carbon Nanotubes and Carbon Fibers

**DOI:** 10.3390/ma15228055

**Published:** 2022-11-15

**Authors:** Muhammad Usama Salim, Farzana Mustari Nishat, Taekgeun Oh, Doo-Yeol Yoo, Yooseob Song, Togay Ozbakkaloglu, Jung Heum Yeon

**Affiliations:** 1 Materials Science, Engineering, and Commercialization Program, Texas State University, San Marcos, TX 78666, USA; 2 Civil Engineering Program, Ingram School of Engineering, Texas State University, San Marcos, TX 78666, USA; 3 Department of Architectural Engineering, Hanyang University, Seoul 04763, Republic of Korea; 4 Department of Civil and Environmental Engineering, University of Alabama in Huntsville, Huntsville, AL 35899, USA

**Keywords:** electrical heating, cementitious composites, carbon nanotubes, carbon fibers, electrical resistivity, mechanical properties

## Abstract

This study investigates the electrical heating (also known as Joule heating) characteristics of cementitious composites containing multi-walled carbon nanotubes (CNT) and carbon fibers (CF) as electrically conductive media in an attempt to develop an eco-friendly and sustainable solution to snow and ice removal on roadway pavements during the winter season. Various dosages of CNT and CF between 0 and 1.0% (by weight of cement) were tested to find the optimum mixture proportions that yield high-energy and efficient electrical-heating performance with superior mechanical properties. The electrical properties were characterized by measuring the electrical resistivity and temperature rise when attached to a power source. Furthermore, this study examined how the crack width affects the electrical resistivity of cementitious composites containing CNT and/or CF. Compressive and flexural strengths were also measured at different ages of 1, 3, 7, and 28 days to identify how the additions of CNT and CF affect the mechanical properties. Results have shown that adding CF in combination with CNT substantially reduces the electrical resistivity and, in turn, improves the heating performance, as CFs further densify the electrically conductive network in the hydrated matrix; adding either CNT or CF alone was not an effective option to enhance the electrical characteristics. The findings of this study are expected to provide essential information for the design and construction of an electrically heated concrete pavement system with promoted energy efficiency, which will offer a promising solution to enhance winter road maintenance, improve public safety, and provide substantial social cost savings.

## 1. Introduction

According to the nationwide icy-road accident statistics by the Federal Highway Administration of the US Department of Transportation, the icy-road fatality count is 3.6 times the total number of deaths from all other weather hazards [1]. The annual average deaths, injuries, and crashes on icy roads were reportedly 1836, 136,309, and 536,731, respectively, between 2005 and 2014 [2]. In addition, it was reported that a vehicle’s braking distance on icy roads could be increased by up to 4.4 times compared to that on dry roads [3]. Presently, the most accepted winter road maintenance practice across the nation is snowplowing and spraying deicing chemicals, including sodium chloride, calcium chloride, magnesium chloride, ammonium nitrate, urea, and calcium magnesium acetate [4]. However, applying deicing chemicals is time-consuming, labor-intensive, quality-sensitive, and environmentally unfriendly. This could lead to the pollution of surface, groundwater, wetland, agriculture, vegetation, wildlife, and soil, and the salinization of lakes, rivers, and streams [5]. Moreover, the use of deicing chemicals has been claimed to be a major cause of concrete infrastructure damages in the form of surface scaling [6] and rebar corrosion [7], as well as corrosion of vehicles [8]. Accordingly, alternatives to melting ice and snow on paved surfaces during the winter season with a less environmental impact and higher effectiveness have been necessitated in the past few decades.

Several active heating technologies have been proposed to develop an eco-friendly and sustainable solution to snow and ice removal for roadways, airport pavements, and other surfaces to achieve a safe, accessible, and operational transportation network during the winter season, including conductive heating, induction heating, heating cables and pipes, microwave heating, and hydronic heating. The heated pavement system has the characteristic of being heated when electrical power or a magnetic field is supplied. It is considered an environmentally friendly and cost-effective method since it saves on plow operation, deicing chemicals, and wastewater treatment of chemical runoff [4]. Wan et al. reported that using steel slag-based ultra-thin friction courses with steel fiber effectively provided an enhanced induction heating property [9]. Another study by Liu et al. investigated the microwave heating-induced ice-melting performance of asphalt mixtures using activated carbon powder filler [10] and reported that the heating efficiency of asphalt greatly improved as the amount of activated carbon filler increased. Moreover, Li et al. proposed a novel method to deice asphalt concrete pavement via a conductive wearing course using graphite, carbon fiber (CF), and an epoxy resin mixture [11]. Kim et al. adopted carbon nanotubes (CNT) as a conductive medium to formulate electrically heated concrete mixtures [12] and characterized their heating performance upon external power supply. Wu et al. developed a three-phase composite conductive concrete for pavement deicing using graphite, steel fiber, and CF [13]. Their study reported that the developed composite presented a satisfactory potential for snow-melting during the winter. Garcia et al. studied the electrical heating of asphalt mortar with different volumes of conductive fibers and fillers [14]. A study by Gomis et al. examined the feasibility of using self-heating and conductive cement with carbonaceous materials and modeled their performance [15]. In addition, Sassani et al. and Nahvi et al. devised CF-based electrically conductive concrete to design and construct environmentally friendly deicing pavements and implemented the developed system at Des Moines International Airport [16,17,18].

Although many studies are underway to develop smart, sustainable, and energy-efficient deicing pavement materials and systems, little effort has been made to develop a high-energy and efficient conductive material utilizing CNT and CF. Since CNT is a nanoscale filler that can fill the gaps between macros CFs, if used in a combined form, it will give rise to a substantial improvement in electrical conductivity, which will, in turn, significantly enhance the heating efficiency. In this study, a high-energy and efficient conductive-cementitious composite is designed based on CNT and CF, and its electrical properties, heating performance, and mechanical characteristics are extensively evaluated based on a series of laboratory tests. Cement mortar was used as a test material because the present study is a preliminary work to find optimum combinations of conductive media (CNT and CF) that result in the most effective heating performance. The target temperature range to be achieved upon heating for winter maintenance is set at approximately 5 °C but will vary depending on the climate conditions, such as snowfall intensity, average daily minimum temperature, and wind speed. The research outcomes are expected to provide an effective solution for enhanced winter road maintenance, improved public safety, and substantial social cost savings.

## 2. Materials and Methods

### 2.1. Materials

#### 2.1.1. Cement

ASTM C150 Type I Portland cement was used as a binder, whose chemical compositions and physical properties are summarized in Table 1.

**Figure 1:**
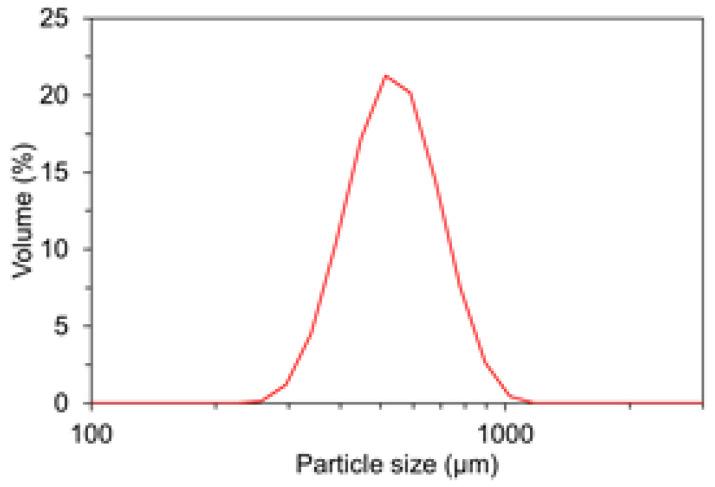
Gradation curves for fine aggregate.

The phase compositions of the cement estimated as per the Bogue calculation were 60.65% C3S, 10.72% C2S, 9.22% C3A, and 8.82% C4AF. Standard sand with a specific gravity of 2.65, fineness modulus of 2.87, the absorptivity of 1.02%, and SiO_2_ content of 98.4% was used as a fine aggregate. The median diameter (D50) was measured to be 533 mm based on the particle size analysis. Figure 1 presents the particle size distribution of the standard sand used.

#### 2.1.2. Electrically Conductive Media

**Figure 2:**
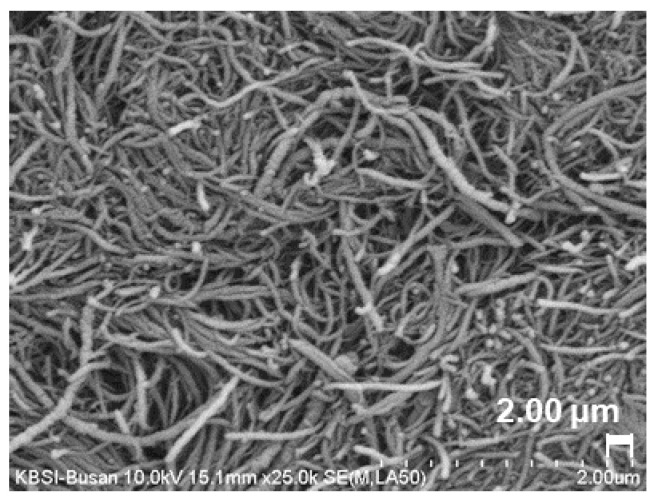
SEM image of the dry CNT used in this study (×25.0k).

A chemically functionalized multi-walled CNT (K-Nanos SWT300; Kumho Petrochemical, Seoul, Republic of Korea) was used as the primary conductive medium to impose electrical conductivity in the cementitious composites. The CNT used in this study was a water-based suspension in which CNT was dispersed through a sodium dodecyl sulfate-based chemical surfactant. The chemical surfactant was used to uniformly distribute the CNT in the matrix. Because CNT particles are under strong Van der Waals forces, they tend to entangle and remain closely packed. Chemical surfactants are known to effectively reduce the Van der Waals forces and help to homogeneously disperse the CNTs in the matrix without damaging the fresh and hardened properties of cementitious composites [19,20]. Many previous studies revealed the effectiveness of chemical surfactants in uniformly dispersing nanoparticles in the matrix [21,22]. The CNT concentration of the suspension was 3.0%. The viscosity, surface resistance, and pH of the suspension were <300 cps, <300 (Ω/sq.), and 7–8, respectively. The CNT dissolved in the solution was a bundle type with a 2.4–3 µm bundle diameter, 27 µm bundle length, 8–15 µm diameter, >95% purity, 0.80 crystallinity (IG/ID), and 0.015–0.030 g/mL bulk density. Figure 2 shows the scanning electron microscope (SEM) image of the CNT used (×25.0k).

**Figure 3:**
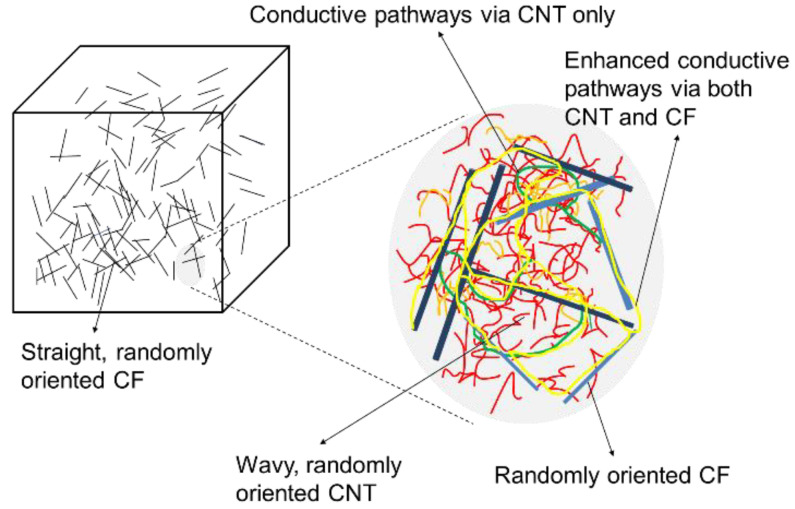
Conceptual schematics that present improved electrically conductive pathways using CNT and CF simultaneously.

As the supplementary electrically conductive medium, polyacrylonitrile (PAN)-based unsized CFs chopped with a length of 6 mm were employed. The rationales for choosing unsized 6-mm macro-CFs as the conductive medium were: (1) to maximize the contacts between the electrical media in the matrix (i.e., CF filaments and CNT particulates), as illustrated in Figure 3, and (2) to provide the electrical continuity across cracks by the bridging effect, which prevents malfunctioning of the electrically heated pavement system upon cracking. It is also well known that PAN-based CFs have relatively high carbon yield and a thermally stable, highly oriented molecular structure [23]. Moreover, some previous studies that used the chopped 6-mm CF as an electrically conductive medium obtained favorable results [24,25].

**Figure 4:**
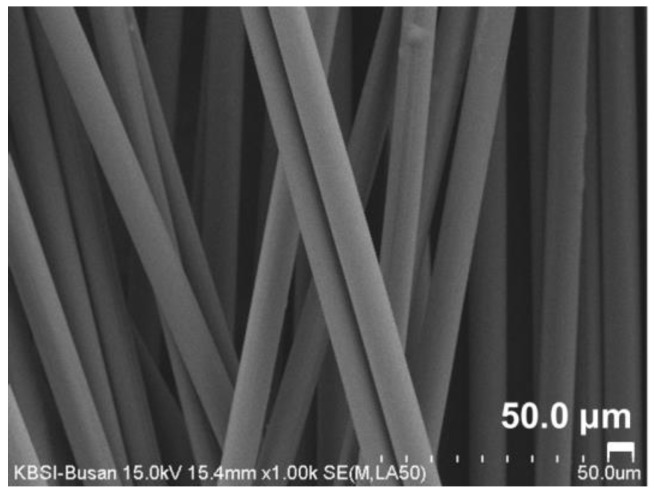
SEM image of the unsized CF used in this study (×1.0k).

The CF used in the present study had a carbon content of 95%, electrical resistivity of 1.54 × 10^−3^ Ω·cm, maximum elongation of 2.3%, a tensile strength of 4810 MPa, a tensile modulus of 225 Gpa, bulk density of 1.78 g/mL, and filament diameter of 6.97 µm. Figure 4 shows the SEM image of the CF used in this study (×1.0k).

### 2.2. Methods

#### 2.2.1. Effect of CNT and CF Additions on the Electrical Resistivity

The electrical resistivity was measured using cube specimens with dimensions of 50 mm × 50 mm × 50 mm. The amount of CNT and CF varied from 0 to 1.0% (by mass of cement) at a 0.5% interval to assess the effect of CNT and CF amounts on the electrical resistivity. For each mixture, two replicates were tested to ensure the reliability of the data. Table 2 shows the mixture proportions used in this study.

A fixed water-to-cement (*w/c*) ratio of 0.45 and a fine aggregate-to-cement (*a/c*) ratio of 2.4 were used. The water content was adjusted per the free water included in the CNT suspension (97% by mass) to keep the effective *w*/*c* of 0.45. A 5-L Hobart mixer (HD-111; Hyundai Precision Industry Co. Ltd., Gwangju, Republic of Korea) was used for mixing as per the standard procedures. CFs were added to the mixture at the last stage.

**Figure 5:**
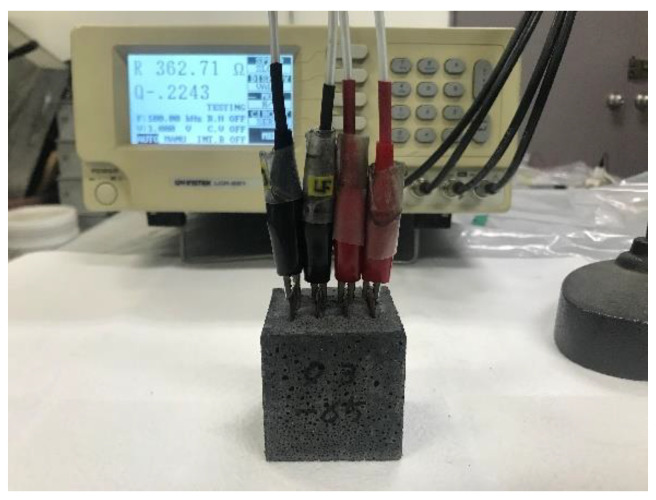
Electrical resistivity measurement using an LCR meter.

For electrical resistivity measurements, the Wenner 4-probe method was used because the 2-probe method that adopts a direct current (DC) voltage source may result in polarization of the ions in the pore solution and, in turn, generate unstable and inaccurate output signals [26]. Moreover, since the output signals collected by the 4-probe method are free from the influence of contact resistance between the electrode and surrounding material, the 4-probe method tends to provide more reliable test results than the 2-probe method. To implement the 4-probe method, an LCR meter (LCR-821; GW Instek, Taipei, Taiwan) operating on an alternating current (AC) voltage source and configured with an electric circuit layout involving four probes was used, as shown in Figure 5.

Four copper plates with dimensions of 25 mm (W) × 50 mm (H) × 0.8 mm (T) were inserted at a constant spacing of 10 mm with an insertion depth of 40 mm to provide a connection platform between the specimen and LCR meter. Because the measured output by the LCR meter is an electrical resistance of the specimen, it was converted to an electrical resistivity as follows:

(1) $$\rho =R\cdot \frac{A}{l}$$

where ρ is the electrical resistivity (Ω·m), R is the electrical resistance (Ω), A is the cross-sectional area of the electrode (m^2^), and l is the linear spacing between the electrodes (m).

**Figure 6:**
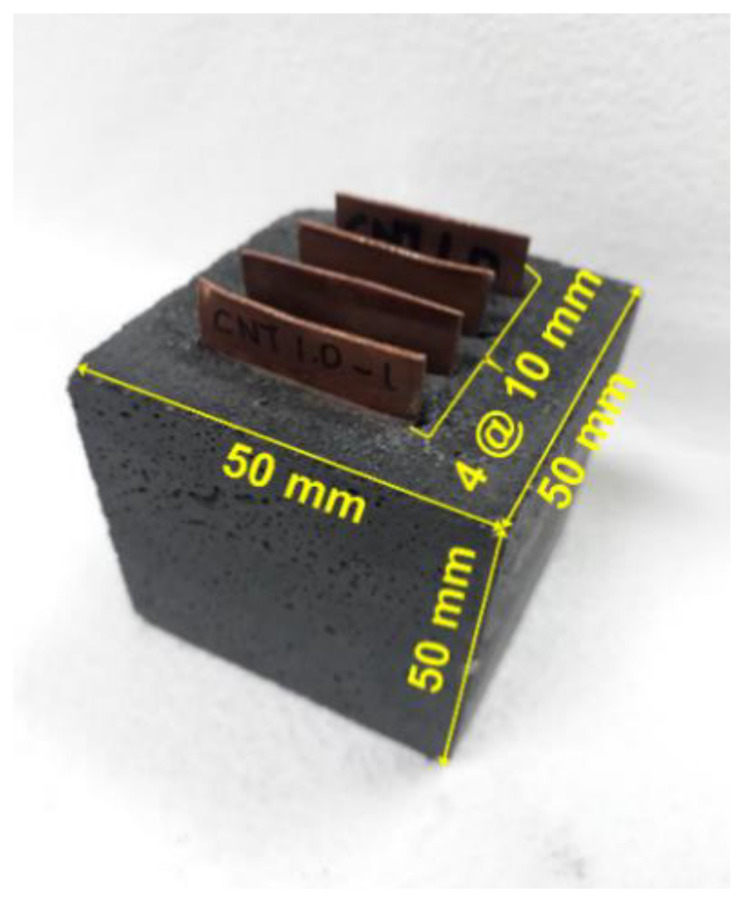
Specimen embedded with four electrodes for electrical resistivity measurement.

Figure 6 presents the specimen used for measuring the electrical resistivity. The electrical resistivity of each specimen was measured for 28 days.

Between each measurement, all the specimens were stored at a constant temperature of 30 ± 1 °C and relative humidity (RH) of 40 ± 1% to accelerate drying because the electrical resistivity depends on not only the connectivity of the electrically conductive medium (i.e., contact/tunneling conductivity) but also the internal moisture state (i.e., ionic conductivity) [27,28,29]; by drying the specimens, the contribution by the electrically conductive medium can be signified from the measured electrical resistivity.

#### 2.2.2. Effect of Apparent Crack Width on the Electrical Resistivity with CNT and CF Additions

**Figure 7:**
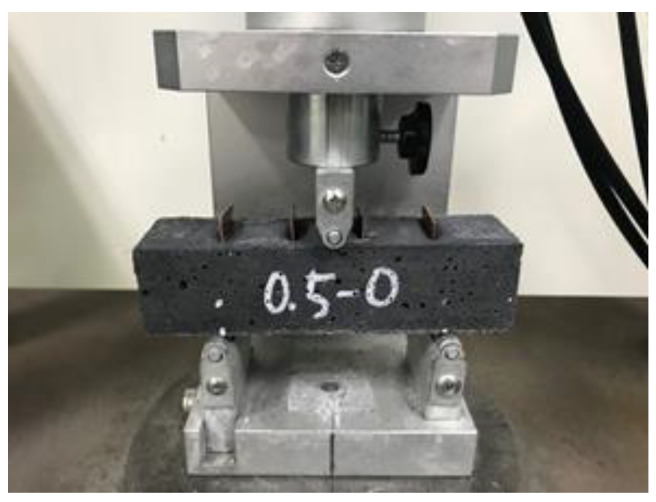
Induction of flexural crack at the mid-span of the specimen.

To assess the CF effect of crack width on the electrical resistivity of cementitious composites randomly distributed with chopped CF and CNT along the crack interface, 40 mm × 40 mm × 160 mm prismatic specimens were fabricated with two different levels of CNT (0.5 and 1.0%) and three different levels of CF (0, 0.5, and 1.0%), i.e., CNT0.5-CF0, CNT1-CF0, CNT1-CF0.5, and CNT1-CF1. To measure the electrical resistivity across a crack via the 4-probe method, four 25 mm-wide electrodes were situated at a constant spacing of 30 mm with an insertion depth of 30 mm. After 72 h of moist curing, each specimen was loaded at a very low load rate of 10 N/s to induce a crack at the mid-span of the specimen (between the two electrodes in the middle), as shown in Figure 7.

**Figure 8:**
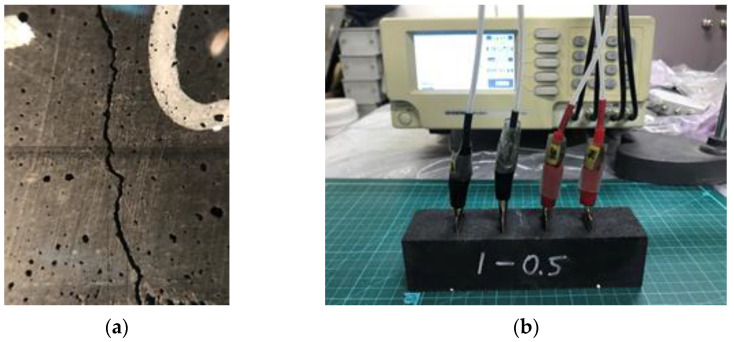
Electrical resistivity measurements for different apparent crack widths: (**a**) crack width control using a crack-measuring microscope; and (**b**) electrical resistivity measurement via the 4-probe method.

As soon as flexural cracking occurred, the specimen was unloaded to keep the CFs not being fully pulled out from the matrix. Subsequently, the electrical resistivity was measured while controlling the crack width as desired using a crack-measuring microscope and a clamp, as indicated in Figure 8.

#### 2.2.3. Joule Heating Characteristics with CNT and CF Additions

**Figure 9:**
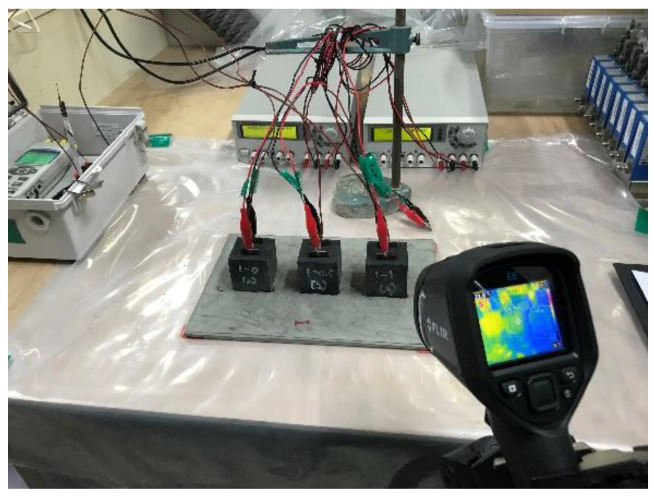
Internal and surface temperature measurements via a thermocouple and infrared thermal analyzer upon voltage supply.

Cube specimens (50 mm × 50 mm × 50 mm) embedded with two 25-mm spaced electrodes were fabricated using the identical mixture proportions used in the electrical resistivity test. The insertion depth of the electrodes was 40 mm. A DC power supply (OPE-503QI; ODA Technology Inc., Incheon, Republic of Korea) was used to apply an electrical potential; the maximum output voltage and current of the power supply were 50V and 3A, respectively (150 VA). At 28 days, an electrical potential of 8V DC was applied between the two electrodes embedded to induce the Joule heating effect; for those whose temperature rise was insignificant under 8V DC (i.e., CNT0-CF0, CNT0-CF0.5, CNT0-CF1, and CNT0.5-CF0), additional testing was performed under an electrical potential of 50V DC. Simultaneously, the Joule heating-driven inner temperature rise and the surface temperature changes were measured using a T-type thermocouple installed at the core of the specimen and an infrared thermal analyzer (FLIR E8; Teledyne FLIR, Wilsonville, OR, USA), respectively, as shown in Figure 9.

The temperature monitoring/scanning was done every 15 s while maintaining the ambient temperature at 22 ± 1 °C. The temperature measurement was finished either when the internal temperature reached 70 °C or when the run time reached 30 min. An adjustable tripod was used to capture the thermal images at a fixed position.

#### 2.2.4. Flexural and Compressive Strengths

Three parallel prism specimens with dimensions of 40 mm × 40 mm × 160 mm were fabricated using the mixture proportions previously given in Table 2. After 24 h of casting, all the specimens were demolded and then cured at 23 ± 0.5 °C and 98 ± 1% RH until the specified ages were reached, i.e., 1, 3, 7, and 28 days. The flexural strength was measured as per ASTM C348 (Standard Test Method for Flexural Strength of Hydraulic-Cement Mortars) using a center-point loading with a constant load rate of 50 N/s. The compressive strength was measured in accordance with ASTM C349 (Standard Method for Compressive Strength of Hydraulic-Cement Mortars Using Portions of Prisms Broken in Flexure) using two portions of the prisms broken in flexure. A constant load rate of 2400 N/s was applied onto the load-bearing area of 40 mm × 40 mm until complete failure using a 200-kN servo hydraulic universal testing machine (UTM). The compressive and flexural strengths were determined before heating after being cured in standard conditions.

## 3. Results

### 3.1. Effect of CNT and CF Additions on the Electrical Resistivity of Cementitious Composites

**Figure 10:**
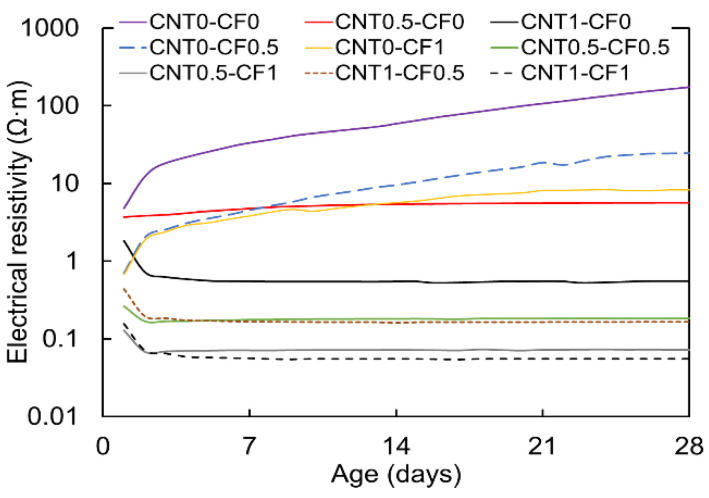
Time-dependent changes in electrical resistivity with various CNT and CF combinations.

Figure 10 presents how the CNT and CF additions affect the electrical resistivity over time.

It is evident from the results that the electrical resistivity substantially declined with more CNT and CF additions. The improved electrical conductivity (reduced electrical resistivity) with increased additions of CNT and CF is because more conductive media dispersed in the matrix create a greater number of contacts among them, which effectively enhances the electrical conduction via the contact and tunneling mechanisms [28,30]. Particularly, it is interesting to note that the specimens with more than 0.5% CNT addition (i.e., CNT0.5-CF0, CNT1-CF0, CNT0.5-CF0.5, CNT0.5-CF1, CNT1-CF0.5, and CNT1-CF1) showed significantly lower electrical resistivity than those with less than 0.5% CNT and with CF only (i.e., CNT0-CF0, CNT0-CF0.5, and CNT0-CF1).

Additionally noted is that the electrical resistivity was even further reduced when CF was used in combination with CF rather than incorporating CNT only. These findings indicate that the addition of nanoscale CNT particles was quite effective in forming a more densely connected electrically conductive network by filling the gaps among macroscale CFs. Moreover, it is noteworthy that the electrical resistivity of the specimens with more than 0.5% CNT was kept almost constant or reduced after the induction period, while the other specimens without CNT substantially increased over time. This finding is because the CNT network was fully percolated at a 0.5% addition, and the electrical resistivity was no longer affected by the ionic conduction mechanism, only by the contact and tunneling conduction [28]. The reason for the tremendous increase in electrical resistivity with age for the non-CNT group (i.e., CNT0-CF0, CNT0-CF0.5, and CNT0-CF1) was because they were under-percolated, and at the same time, continuously lost the moisture from the inside, which eventually reduced the interconnectivity of the electrically conductive network in the matrix. The moisture loss leads to a reduction in electrolytic pore solution that provides lower ionic charge carriers in the matrix that are essential to forming electrically conductive pathways in cementitious composites [27,28,29]. In addition, Han et al. reported that the field emission effect of carbon fiber-based composites reduces when water evaporates from the matrix, leading to an increased electrical resistivity [31].

Lastly, another important finding from Figure 10 is that the effect of CNT was more prominent in reducing the electrical resistivity than CF when the same amount (by weight) was added. For instance, when CNT0-CF1 and CNT1-CF0 were compared, CNT1-CF0 showed 0.7 times less electrical conductivity, while CNT0-CF1 showed 34.5 times more electrical conductivity at 28 days. The reason for the enhanced conductive nature of CNT is attributed to its very tiny size and interconnected tunnel pattern in the matrix. Above all, it is of great importance to note that combining CNT and CF provided the most efficient results.

### 3.2. Effect of CNT and CF Additions on the Joule Heating Characteristics of Cementitious Composites

**Figure 11:**
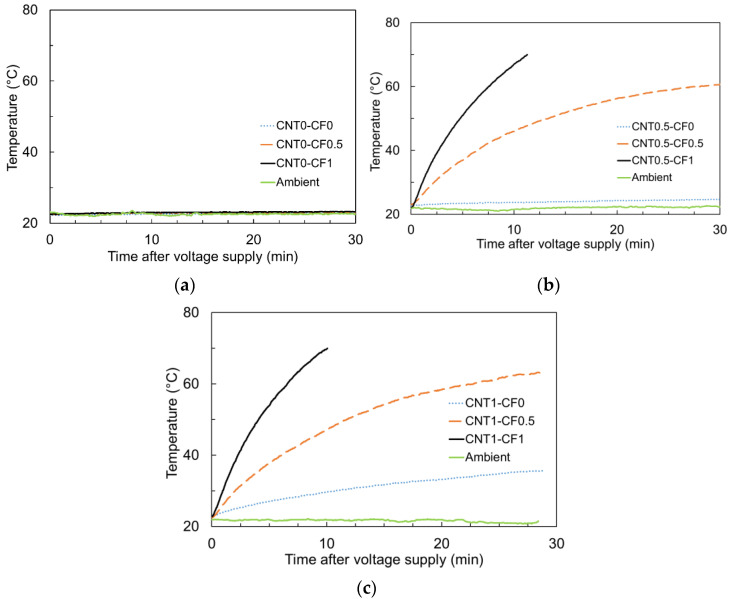
Joule-heating-driven temperature rise of cementitious composites: (**a**) 0% CNT series, (**b**) 0.5% CNT series, and (**c**) 1% CNT series upon 8V DC supply.

Figure 11a–c illustrates the heating curves for 0, 0.5, and 1% CNT groups upon 8V DC, respectively. Overall, better heating performance was observed as the electrical resistivity decreased, which is in good agreement with former studies [9,10,11,12,13,14,15,16,17,18].

This is because Joule’s law of heating is given by the product of its resistance and the square of the current; thus, if the electrical resistance is reduced by a factor of 1/*n*, the power of heating becomes *n* times higher under a constant voltage [12,32]. It is apparent from Figure 11a that the specimens with CF alone showed a negligible heating performance because they had approximately 15 to 310 times higher electrical resistivity than that of CNT1-CF1 (the specimen with the lowest electrical resistivity).

**Figure 12:**
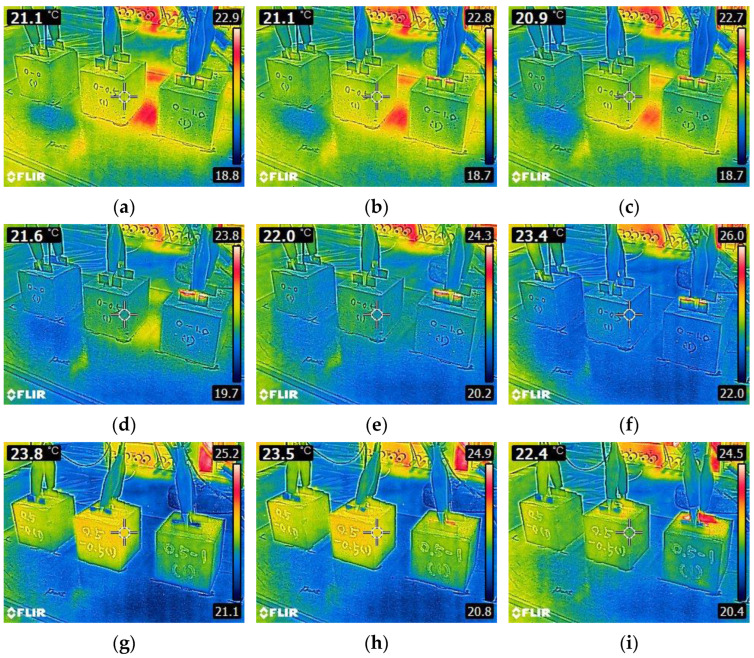
Thermal images of 0% CNT series specimens (CNT0-CF0, CNT0-CF0.5, and CNT0-CF1, respectively, from the left) taken at: (**a**) 0 s, (**b**) 45 s, (**c**) 90 s, (**d**) 135 s, (**e**) 180 s, (**f**) 225 s, (**g**) 270 s, (**h**) 315 s, and (**i**) 360 s upon 8V DC supply.

This was also obvious from Figure 12, showing the surface temperature profile of CNT0-CF0, CNT0-CF0.5, and CNT0-CF1 upon 8V DC. Note that there were little changes in the surface temperature during the first 360 s of power supply due to their significantly higher electrical resistivity.

**Figure 13:**
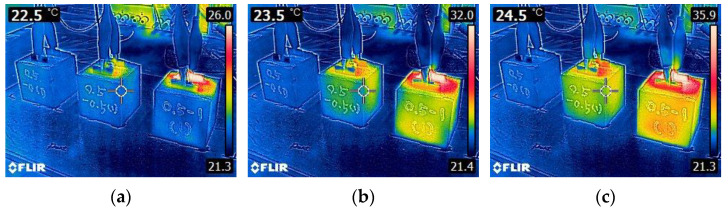

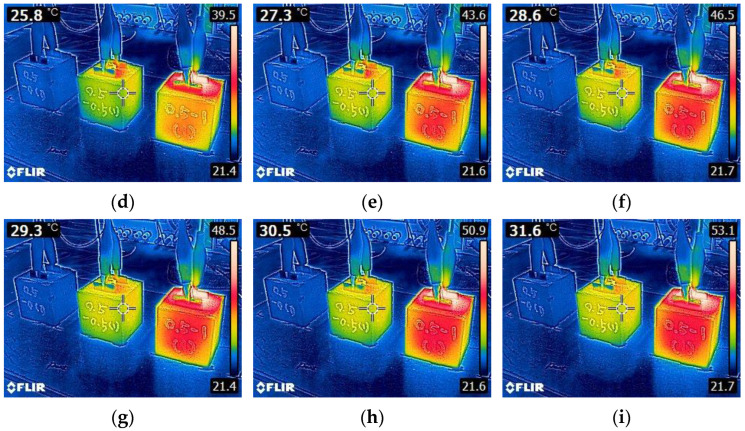
Thermal images of 0.5% CNT series specimens (CNT0.5-CF0, CNT0.5-CF0.5, and CNT0.5-CF1, respectively, from the left) taken at: (**a**) 0 s, (**b**) 45 s, (**c**) 90 s, (**d**) 135 s, (**e**) 180 s, (**f**) 225 s, (**g**) 270 s, (**h**) 315 s, and (**i**) 360 s upon 8V DC supply.

However, as can be seen in Figure 11b, the specimens showed a substantial heating behavior even upon 8V DC when 0.5% CNT was added, particularly when used in combination with CF. Whereas CNT0.5-CF0 exhibited only a few degrees higher temperature than the ambient temperature, CNT0.5-CF0.5 and CNT0.5-CF1 showed dramatic deviations from the ambient temperature. The highest temperature monitored was 70 °C for CNT0.5-CF1 (early termination of the heating test after 10 min as the specimen reached 70 °C), 60.7 °C for CNT0.5-CF0.5, and 24.7 °C for CNT0.5-CF0. This finding again proves that when CNT and CF were used together, the electrical resistivity was significantly reduced, leading to more significant heat generation. The surface temperature profiles of the 0.5% CNT group are provided in Figure 13. It was evident from the thermal images that more CF additions resulted in a much faster and higher temperature rise under the same level of electrical potential.

**Figure 14:**
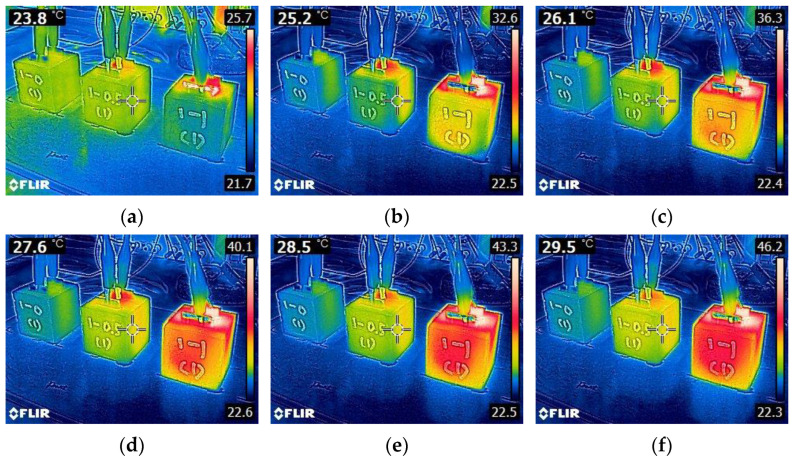

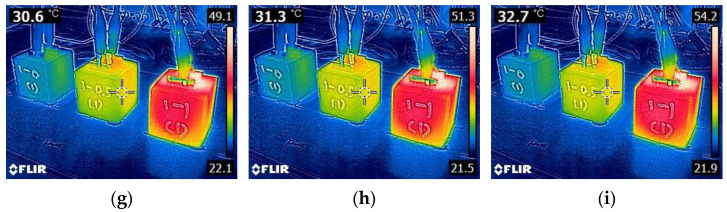
Thermal images of 1% CNT series specimens (CNT1-CF0, CNT1-CF0.5, and CNT1-CF1, respectively, from the left) taken at: (**a**) 0 s, (**b**) 45 s, (**c**) 90 s, (**d**) 135 s, (**e**) 180 s, (**f**) 225 s, (**g**) 270 s, (**h**) 315 s, and (**i**) 360 s upon 8V DC supply.

Additionally, Figure 11c presents the heating curves for the specimens with a 1% CNT and varying percentages of CF (i.e., 0, 0.5, and 1% by weight of cement). The result indicates that the specimens with 1% CNT showed a quicker and higher temperature rise as compared to those with 0.5% CNT. It is especially interesting to note that CNT1.0-CF0 showed approximately five times greater temperature rise than CNT 0.5-CF0 after 30 min of power supply as much denser electrically conductive networks were created as more CNT was added. Also noted was, similarly to the results observed in the 0.5% CNT group, using CF tended to remarkably increase the electrical conductivity and resulting heating performance. This finding is attributed to the formation of more stable conductive pathways in the hydrated matrix using macroscale CFs, which further facilitated the flow of electric current [11]. The surface temperature profiles of CNT1-CF0, CNT1-CF0.5%, and CNT1-CF1 for the first 360 s after 8V DC supply are given in Figure 14.

**Figure 15:**
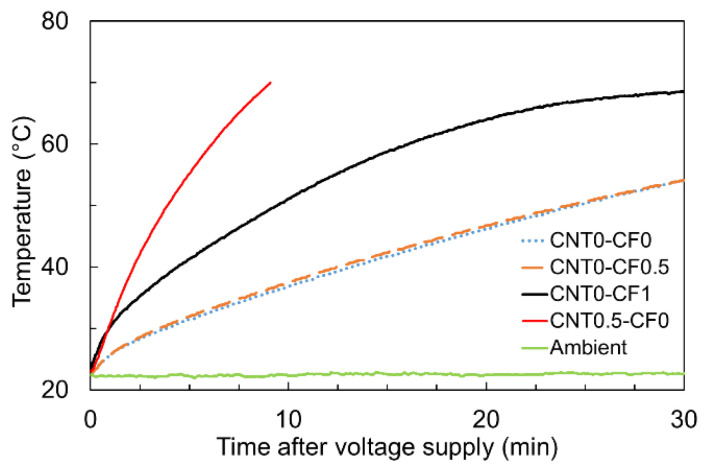
Joule-heating-driven temperature rises of CNT0-CF0, CNT0-CF0.5, CNT0-CF1, and CNT0.5-CF0 specimens upon 50V DC supply.

Lastly, Figure 15 presents the heating curves measured from a group of specimens that provided little temperature increase under 8V DC (i.e., CNT0-CF0, CNT0-CF0.5, CNT0-CF1, and CNT0.5-CF0) when a higher level of electrical potential (50V DC) was applied for 30 min.

**Figure 16:**
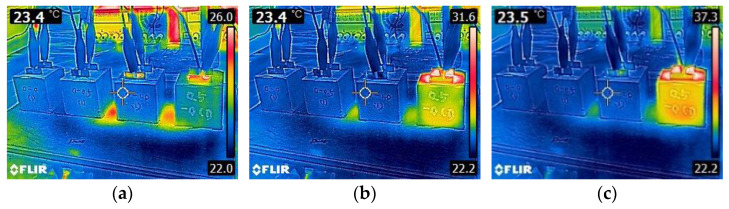

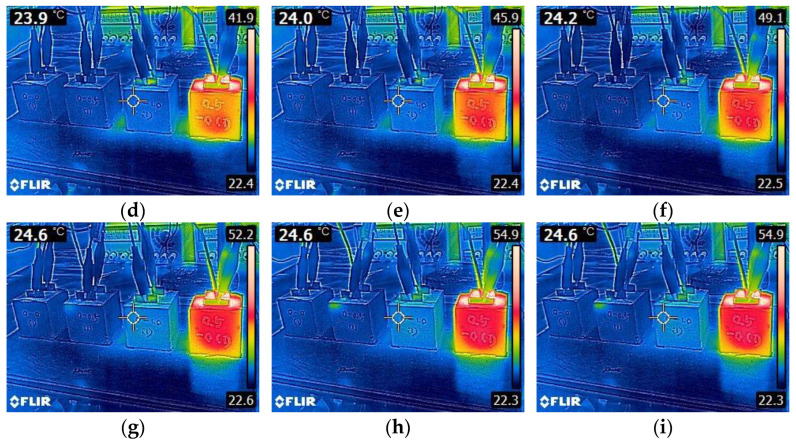
Thermal images of CNT0-CF0, CNT0-CF0.5, CNT0-CF1, and CNT0.5-CF0 (from the left) specimens taken at (**a**) 0 s, (**b**) 45 s, (**c**) 90 s, (**d**) 135 s, (**e**) 180 s, (**f**) 225 s, (**g**) 270 s, (**h**) 315 s, and (**i**) 360 s upon 50V DC supply.

Note that CNT0.5-CF0 showed significantly better heating performance compared to the specimens with CF only, which was in good agreement with the result of electrical resistivity measurements previously discussed; the 28-day electrical resistivity of CNT0-CF0, CNT0-CF0.5, and CNT0-CF1 was found to be 30.6, 4.3, and 1.5 times higher than that of CNT0.5-CF0, respectively. The surface temperature profiles of CNT0-CF0, CNT0-CF0.5, CNT0-CF1, and CNT0.5-CF0 under 50V DC are displayed in Figure 16, which coincided well with the temperature measurements depicted in Figure 15.

### 3.3. Effect of Apparent Crack Width on the Electrical Resistivity of Cementitious Composites with CNT and CF Additions

**Figure 17:**
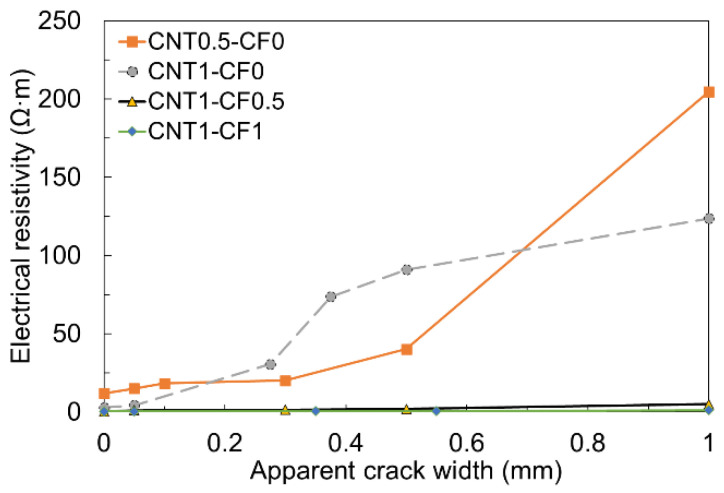
Effect of apparent crack width on the electrical resistivity with various CNT and CF combinations.

Figure 17 presents how the crack width affects the electrical resistivity of cementitious composites.

Four different specimens (i.e., CNT0.5-CF0, CNT1-CF0, CNT1-CF0.5, and CNT1-CF1) were tested to see how CNT, CF, and their combination, affected the electrical resistivity under a controlled crack width of up to 1 mm. When only CNT was added (i.e., CNT0.5-CF0 and CNT1-CF0), the electrical resistivity dramatically increased with an increase in crack width, regardless of the CNT content. Particularly, a notable jump in electrical resistivity was observed when the crack width became wider than 0.3 mm. The reason can be attributed to the rupture of the electrical conduction paths upon the occurrence of a crack [33]; because the size of the CNT used in this study was only up to 1/10^6^ of the apparent crack width, CNT no longer provided effective electrical paths across the crack, which subsequently resulted in immensely higher electrical resistivity. However, when up to 1% CF was added in combination with CNT, little increase in the electrical resistance was noted even when the crack width was as wide as 1 mm. This is because 6-mm long CF filaments provided a sufficient bridging effect at the crack interface, allowing the electrons to pass across the crack.

The findings provide an important practical implication that the sole use of CNT might not be viable for infrastructure applications that require a continuous electrical power supply, such as an electrically heated pavement system, because cracks will eventually lead to the malfunction of the system; the use of CFs with a sufficient fiber length could be an effective and practical solution as they could link the crack interfaces, which helps the system keep functioning even after cracking. On the other hand, the crack-sensitive electrical response of the sole CNT system could be rather beneficially utilized to detect failures and cracks in various types of concrete infrastructure (i.e., self-sensing applications).

### 3.4. Effect of CNT and CF Additions on the Compressive and Flexural Strengths of Cementitious Composites

**Figure 18:**
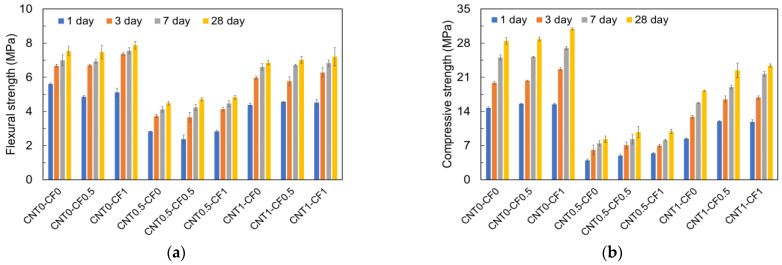
Effect of CNT and CF additions on the strengths: (**a**) flexural strength; (**b**) compressive strength.

Figure 18 compares the compressive and tensile strengths of cementitious composites with different additions of CNT and CF measured at 1, 3, 7, and 28 days.

The general trend was quite similar between the compressive and tensile strengths at all ages. First, it is noted that the addition of CNT adversely affected the mechanical properties at a lower addition (0.5%). Morsy et al. reported a similar finding that the compressive strength of concrete decreased when CNT was added by more than 0.2% by weight of cement [34]. Another reason for the strength reductions with CNT additions may be the utilization of non-vacuumed CNT suspension with a chemical surfactant to disperse the CNT in water uniformly. Targazikis et al. revealed that using a chemical surfactant for CNT dispersion generated numerous air voids in concrete, ultimately leading to a reduction in strength [35]. Moreover, the overdose of a chemical surfactant could be a probable reason that caused the mechanical degradation [36]. Improper dispersion of CNT in the matrix could also be another possibility for the reduced mechanical strengths since the agglomeration of CNT can occur, which forms weak zones in the matrix and weakens the bond between CNT and hydration products [37,38]. Siddique and Mehta associated the strength reductions with microcracks that occurred due to the insufficient wetting of CNT since the CNT subjected to weaker bonding can be ultimately pulled out of the matrix [39].

However, when the CNT addition was increased to 1%, a substantial improvement in the mechanical properties was observed. This finding may be explained by the filler effect. When CNT was added up to 0.5% (under percolated mixture), the air entrainment effect can be stronger than the filler effect and thus can reduce the mechanical strengths. On the contrary, when 1% CNT was used (over-percolated), the filler effect controls the mechanical behavior over the air entrainment effect, which can result in higher compressive and tensile strengths [35,40]. For instance, CNT1-CF0, CNT1-CF0.5, and CNT1-CF1 showed 1.19, 3.75, and 1.36 times higher compressive strengths, and 1.53, 1.49, and 1.49 times higher tensile strengths than CNT0.5-CF0, CNT0.5-CF0.5, and CNT0.5-CF1 at 28 days.

Furthermore, the results revealed a positive effect of CF additions on the mechanical strengths, and the effect became greater as the addition of CF increased. For example, CNT0-CF1 showed 8.7 and 4.4% higher compressive and tensile strengths than the control mix, respectively, at 28 days. These results agreed well with previous studies conducted on the effect of CF on mechanical strength [41,42]. The reason can be ascribed to the higher tensile strength and elastic modulus of CF, which prevented the initiation and propagation of cracks in the matrix. The addition of CF also tended to help the compressive and tensile strength gains when used in combination with CNT. For example, the 28-day compressive and tensile strengths of CNT0.5-CF1 were comparable to or higher than those of CNT0.5-CF0 and CNT0.5-CF0.5, which is in agreement with some of the previous studies [31,34].

The compressive and tensile strengths of all the specimens increased gradually over time, but the CF incorporation tended to contribute to a higher early strength gaining; the more, the higher early strengths were obtained. For instance, when only CFs were added up to 0.5 and 1% without CNT, the specimens showed 2.1 and 14.2% more compressive strengths and 1.0 and 10.3% higher tensile strengths, respectively, during the first 3 days as compared to the control specimen. More than 70% of the 28-day strength was achieved within 3 days. The reason may be attributed to the higher hydration rate in the presence of CFs. On the contrary, CNT incorporations did not help in gaining early strengths. For example, when CNTs were added in the absence of CFs in percentages of 0.5 and 1%, the specimens showed 69.2 and 35.0% lower compressive strengths and 44.2 and 10.5% lower tensile strengths, respectively, with respect to the control.

## 4. Conclusions

In this study, the electrical, heating, and mechanical characteristics of cementitious composites utilizing multi-walled carbon nanotubes (CNT) and carbon fibers (CF) as electrically conductive media were investigated based on a series of laboratory tests. The key findings of this study can be summarized as follows:

1. The more CNTs added, the lower the electrical resistivity obtained. The addition of CF reduces the electrical resistivity further because CF helps create a more stable electrically conductive network in the matrix.
2. In instances of similar levels of electrical potential, the temperature rise becomes quicker and greater as the electrical resistivity decreases. This observation is well described by Joule’s law of heating.
3. Adding combinations of CF with CNT can be an effective means to improve the heating performance in contrast to adding either CNT or CF alone.
4. Adding CF helps maintain continuous electrical pathways across cracks when the crack width is kept tighter than one millimeter.
5. Adding up to 0.5% CNT—chemically dispersed using a surfactant—adversely affects both compressive and flexural strengths because, at such low dosage levels, the effect of the chemical surfactant (i.e., void formation) becomes more dominant than the filler effect offered by CNT particulates. As more CNT is added by up to 1%, however, the strengths can be mostly recovered. Adding CF alone rather improves the compressive and flexural strengths.

This study provided key information for the design and construction of an electrically heated concrete pavement system with improved energy efficiency, which will help enhance the effectiveness of winter road maintenance and improve public safety.

## Figures and Tables

**Table 1:** Chemical compositions and physical properties of Portland cement used.

Chemical Composition (%)								Fineness (m^2^/kg)	Specific Gravity (-)
SiO_2_	Al_2_O_3_	Fe_2_O_3_	CaO	MgO	SO_3_	K_2_O	Na_2_O		
19.7	5.33	2.90	61.5	3.81	2.54	0.86	0.18	370	3.15

**Table 2:** Mixture proportions of cementitious composites with CNT and CF.

Mixture	Effective *w*/*c*	Weight per Unit Volume (kg/m^3^)				
		Cement	Fine Aggregate	Water	CNT Suspension	CF
CNT0-CF0	0.485	597.7	1434.5	269.0	0	0
CNT0-CF0.5	0.485	596.7	1432.1	268.5	0	2.98
CNT0-CF1	0.485	595.7	1429.7	268.1	0	5.96
CNT0.5-CF0	0.485	595.9	1430.2	171.8	99.3	0
CNT0.5-CF0.5	0.485	594.9	1427.8	171.5	99.2	2.97
CNT0.5-CF1	0.485	593.9	1425.4	171.2	99.0	5.94
CNT1-CF0	0.485	594.1	1425.8	75.3	198.0	0
CNT1-CF0.5	0.485	593.2	1423.7	75.1	197.7	2.97
CNT1-CF1	0.485	592.2	1421.3	75.0	197.4	5.92
